# Structural Comparison and Therapeutic Effects on Ulcerative Colitis of Fucoidan and Its Derivative from *Saccharina japonica*

**DOI:** 10.3390/md23110426

**Published:** 2025-11-03

**Authors:** Yanlei Yu, Xiaoshu Jin, Yunjie Zhao, Ningning Wang, Yi Hua, Youmin Ying, Bin Wei, Hong Wang

**Affiliations:** College of Pharmaceutical Science & Collaborative Innovation Center of Yangtze River Delta Region Green Pharmaceuticals, Zhejiang University of Technology, Hangzhou 310014, China; yanleiyu@zjut.edu.cn (Y.Y.);

**Keywords:** fucoidan, ulcerative colitis, structural comparison, gut microbiota

## Abstract

Ulcerative colitis (UC) is a chronic inflammatory bowel disease (IBD) that can lead to intestinal complications and systemic risks, significantly increasing the likelihood of colorectal cancer in individuals with long-term illness. Fucoidan has shown potential in alleviating UC; however, the structure–activity relationship remains challenging. The present study aims to compare fucoidan (CF) and its degraded derivatives (DF) in the prevention and treatment of UC. Structural analysis demonstrated that CF and DF possess similar monosaccharide compositions and sulfation content; however, they differ significantly in molecular weight, with CF measuring 582 kDa and DF 2.3 kDa. Additionally, DF display a lower degree of branching compared to CF. Results from the mouse model demonstrated that both CF and DF can effectively alleviate clinical symptoms of UC; however, the underlying mechanisms of action are likely to differ. Both CF and DF produced comparable improvements in the disease activity index. CF demonstrated superior efficacy in alleviating weight loss and maintaining colon length, whereas DF showed greater benefits in protecting the colonic mucosa and reducing inflammatory infiltration. The gut microbiota analysis indicated that DF was more effective in restoring microbial diversity in UC mice. Both CF and DF were capable of modulating microbial imbalances at the phylum and genus levels, although the specific taxa exhibited differences.

## 1. Introduction

Ulcerative colitis (UC) is a chronic inflammatory bowel disease (IBD) that has shown a global increase in incidence [1]. The clinical symptoms of UC primarily include persistent or recurrent diarrhea, mucoid and bloody stools, abdominal pain, and a sense of urgency accompanied by tenesmus after defecation. Some patients may also experience systemic symptoms such as fever, fatigue, and weight loss, while severe cases can lead to life-threatening complications, including massive bleeding and intestinal perforation [2]. The pathogenesis of UC is complex, involving multiple factors such as immune dysregulation, intestinal barrier dysfunction, and gut microbiota imbalance [3]. Research has demonstrated that the gut microbiota composition in UC patients significantly differs from that of healthy individuals, characterized by a reduced proportion of Bacteroidetes and Firmicutes, and an increased proportion of Proteobacteria and Actinobacteria [4,5,6]. Pharmacological treatment remains the cornerstone of UC management, encompassing several major classes of medications, including aminosalicylates, glucocorticoids, and immunosuppressant [7]. While these medications can help control UC symptoms to some extent, they have notable limitations. Most drugs only alleviate symptoms without curing the disease, necessitating long-term or lifelong use, which may result in serious adverse effects, such as an increased risk of infection and cancer. These limitations have prompted researchers to continuously explore new therapeutic strategies, with natural-sourced treatment options gaining increasing attention due to their potential efficacy and favorable safety profiles.

Natural polysaccharides exhibit a wide range of biological activities, including immune regulation, anti-inflammation, antioxidation, and modulation of gut microbiota. Their safety and non-toxic nature make them promising candidates for the treatment of UC [8]. Fucoidan from brown seaweed, *Saccharina japonica*, which is common seafood in China and Japan, has garnered significant attention due to its complex structural composition and rich sulfation characteristics. Fucoidans consist of L-fucose linked by α-(1 → 3) or α-(1 → 4) bonds, forming the main chain, with side chains that include monosaccharides such as galactose, mannose, and glucose. Typically, the sulfation occurs at the C-2 or C-4 positions of fucose [9,10]. Our previous research discovered that fucoidan derived from *Saccharina japonica* significantly reduces the Firmicutes/Bacteroidetes ratio in the gut microbiota of mice on a high-fat diet, increases the relative abundance of beneficial bacteria, and ameliorates microbiota dysbiosis [11]. Furthermore, a novel fucoidan extracted from *Sargassum fusiforme* composed of fucose and galactose exhibited certain beneficial effects on the disorder of intestinal microbiota [12].

Fucoidan has been shown to exhibit both therapeutic and prophylactic effects against UC [13]. The underlying mechanisms are believed to involve fucoidan’s ability to alleviate DSS-induced UC by reducing oxidative stress damage in the colon and enhancing the integrity of the colonic barrier [14]. Evidence suggests that fucoidan effectively attenuates DSS-induced colitis by suppressing inflammatory responses within the colon, reinforcing gut barrier function, modulating of the gut microbiota, and promoting autophagy [15,16]. Furthermore, fucoidan administration has been linked to the alleviation of UC symptoms as well as the reduction in anxiety- and depressive-like behaviors associated with the gut–brain axis [17]. It is important to emphasize that the bioactivity of fucoidan is highly dependent on its structural characteristics [18]. However, challenges arise due to the complex structure and purity of these polysaccharides, making it very difficult to obtain highly pure fucoidan. Purity assessment depends on structural characterization methods such as monosaccharide composition analysis, molecular weight distribution, FT-IR, and NMR spectroscopy. Moreover, research focusing on the detailed structural analysis of fucoidan and its implications for UC treatment and prevention remains limited.

The present study conducted a structural comparison between fucoidan and its degraded derivative, produced via free radical oxidation, and evaluated their respective effects on the prevention and treatment of DSS-induced UC using a mouse model. The research aimed to investigate the impact of structural characteristics, including molecular weight and branching patterns, and to elucidate the potential underlying mechanisms, thereby providing novel strategies and insights for the prevention and treatment of UC.

## 2. Results

### 2.1. Chemical Properties

The extraction yield of CF was determined to be 1.1 ± 0.04% and the yield of DF degraded from CF was 32.9 ± 0.89%. Their chemical properties were shown in Table 1, the standard deviations were calculated three times for extraction and purification (*n* = 3). CF had a total sugar content of 49.6 ± 1.03%, sulfate group content of 20.2 ± 2.03%, and uronic acid content of 1.78 ± 0.11%. It primarily consisted of fucose, galactose, mannose, and glucose, along with minor amounts of glucuronic acid, galacturonic acid, and xylose. Notably, fucose accounted for 55.86% of the monosaccharide composition. The total sugar content of DF was 74.0 ± 1.58%, while the levels of other constituents remained relatively stable. Monosaccharide composition analysis revealed a slight increase in fucose content to 61.58%, a decrease in mannose proportion.

### 2.2. Molecular Weight Distribution

**Figure 1:**
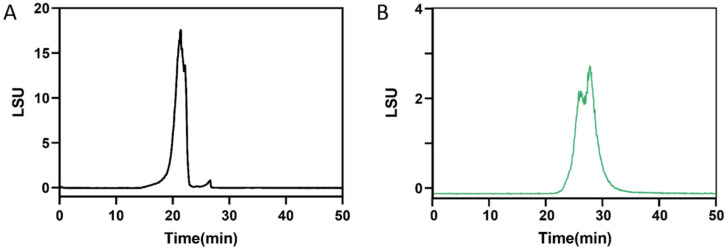
Molecular weight distribution. (**A**) CF determined by TSKgel G5000PWXL connected to G3000PWXL columns, (**B**) DF determined by TSKgel G3000PWXL connected to G-Oligo-PW columns.

The molecular weight distributions of CF and DF were analyzed using G5000 and G3000 columns, respectively. The molecular weight of CF was estimated by comparison with standards of 821.7 kDa, 420.0 kDa, 226.7 kDa, and 123.5 kDa, resulting in a calculated value of 582 kDa (Figure 1A). In contrast, the molecular weight of DF was determined using standards of 21.0 kDa, 12.6 kDa, 4.3 kDa, and 505 kDa, yielding a calculated molecular weight of 2.3 kDa (Figure 1B).

### 2.3. FT-IR Spectrometry

**Figure 2:**
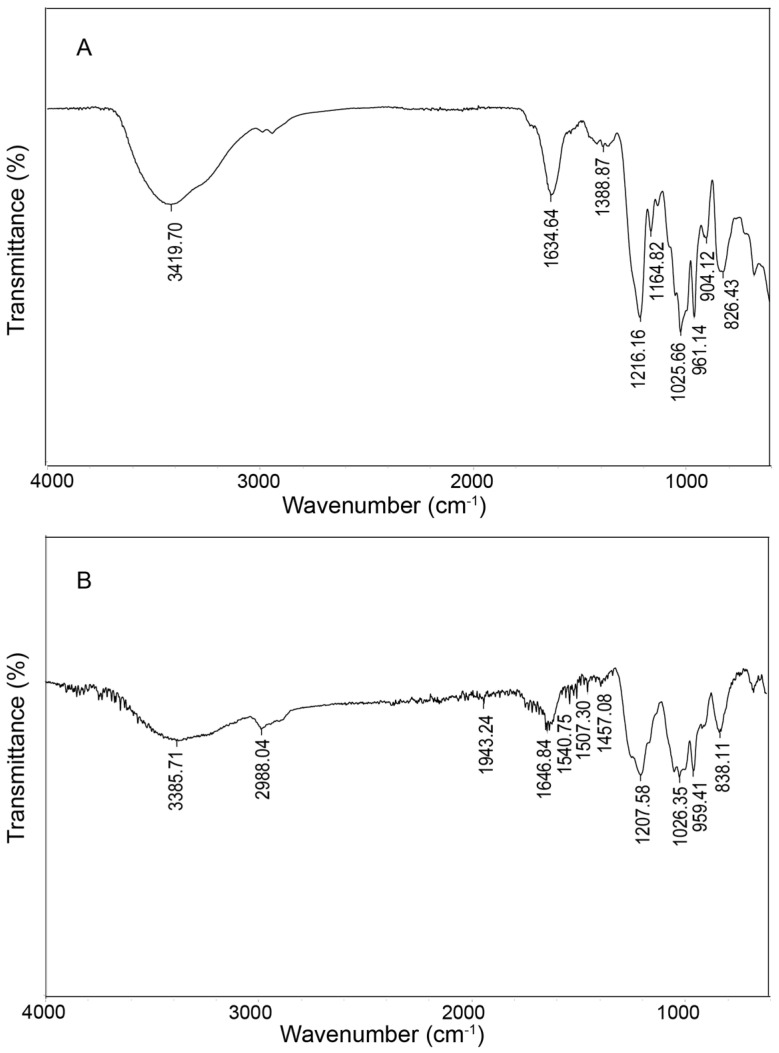
FT-IR analysis of CF (**A**) and DF (**B**).

The FT-IR spectra of CF and DF are presented in Figure 2. In the CF spectrum, the absorption band observed at 3419 cm^−1^ indicates O-H stretching vibrations and the peak at 2989 cm^−1^ corresponds to C-H stretching [19]. The absorption at 1634 cm^−1^ is attributed to C=O stretching vibrations of carboxyl groups present in uronic acids [20]. An absorption at 1388 cm^−1^ arises from C-H bending within the sugar ring structure. The signal at 1216 cm^−1^ is characteristic of S=O stretching vibrations. Additionally, bands at 1164 cm^−1^ and 1025 cm^−1^ are assigned to C-O-H bending and glycosidic C-O-C/C-O stretching vibrations, respectively. The peak at 826 cm^−1^ suggests the presence of α-glycosidic linkages [21]. Similar features were observed in the DF spectrum, including hydroxyl absorption at 3385 cm^−1^, C-H stretching at 2988 cm^−1^, C=O stretching at 1646 cm^−1^, S=O stretching at 1207 cm^−1^, and C-O-C/C-O-H stretching at 1026 cm^−1^.

### 2.4. Glycosidic Linkage Determination by Methylation and GC-MS Analysis

The data obtained from GC-MS were interpreted by comparison with the partially methylated alditol acetates (PMAAs) database and are presented in Table 2. Both CF and DF exhibit a backbone primarily composed of 1,3-linked fucose, constituting 47.25% of CF and 62.28% of DF, with 1,3-linked galactose alternating with the fucose backbone to form the repeating unit → 3)-Fuc*p*-(1 → 3)-Gal*p*-(1→. Branching within the structure is indicated by the presence of 1,3,4-linked fucose and 1,2,3-linked galactose. Notably, DF exhibited a reduction in branching complexity, with the proportion of 1,3,4-Fuc*p* decreasing from 14.62% to 5.82%, and 1,2,3-Gal*p* declining from 10.31% to 4.57%. Furthermore, minor amounts of 1,3-linked mannose and 1,3-linked glucose were detected. Although GlcA and GalA were identified in the monosaccharide composition analysis, their low abundance precluded their detection in the glycosidic linkage profiling.

### 2.5. NMR Spectral Analysis

**Figure A1:**
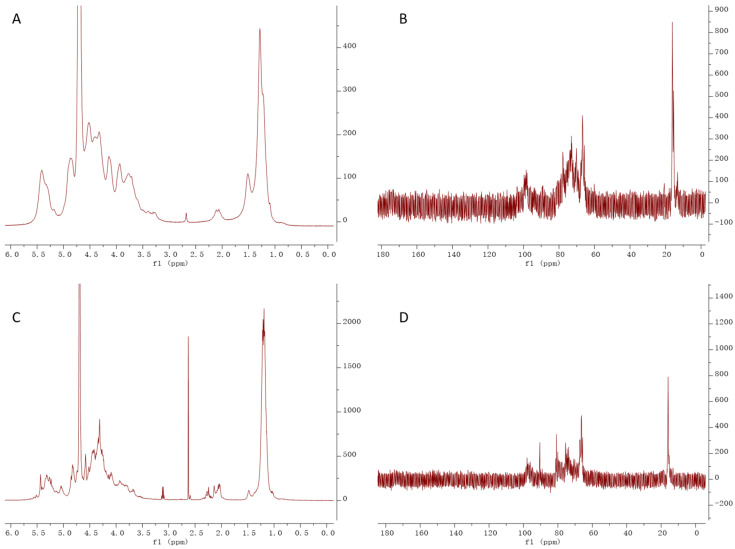
NMR (^1^H and ^13^C) analysis of CF and DF, (**A**) ^1^H NMR of CF, (**B**) ^13^C NMR of DF, (**C**) ^1^H NMR of DF, (**D**) ^13^C NMR of DF.

The ^1^H and ^13^C NMR spectra of CF and DF are shown in Figure A1 (Appendix A.1). The ^1^H NMR spectra of CF and DF primarily exhibited signals in the range of 1.0 to 5.5 ppm, with a D_2_O signal at 4.7 ppm. Proton signals corresponding to the sugar ring were concentrated between 3.2 and 4.5 ppm, while the anomeric proton resonances appeared in the region of 4.5 to 5.5 ppm. The aromeric proton signals at 5.40 ppm for CF and 5.42, 5.31 and 5.03 ppm for DF were assigned to α-L-fucopyranose residues, whereas the high-field signals at 4.52 ppm for CF and 4.53 ppm for DF were attributed to galactopyranose residues [11,12]. Signals observed between 1.2 and 1.4 ppm were assigned to the methyl protons of the fucose residue. In the ^13^C NMR spectra, the anomeric carbon signals of fucose and galactose residues were identified within 95 to 105 ppm. Signals between 60 and 80 ppm were attributed to the C2 to C6 of the sugar residues. Furthermore, a methyl resonance at 16 ppm indicated the C6 of the fucose residue.

### 2.6. Oligosaccharide Profiling of DF

**Figure 3:**
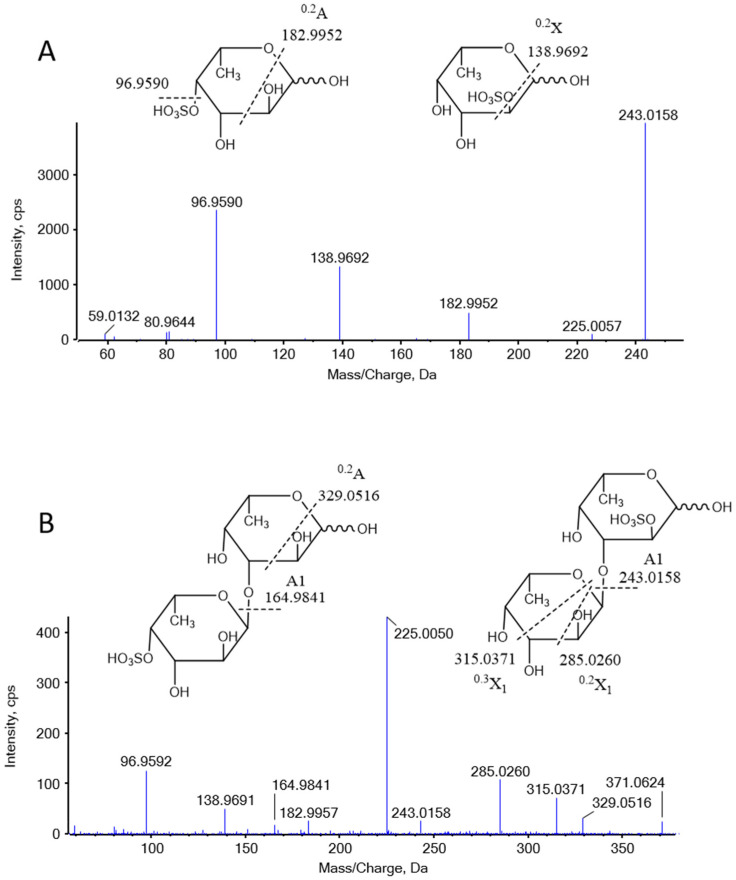
Negative-ion MS/MS product-ion spectra and assignments of the fragments in DF. (**A**) MS/MS analysis of 243 Da, (**B**) MS/MS analysis of 389 Da.

**Figure A2:**
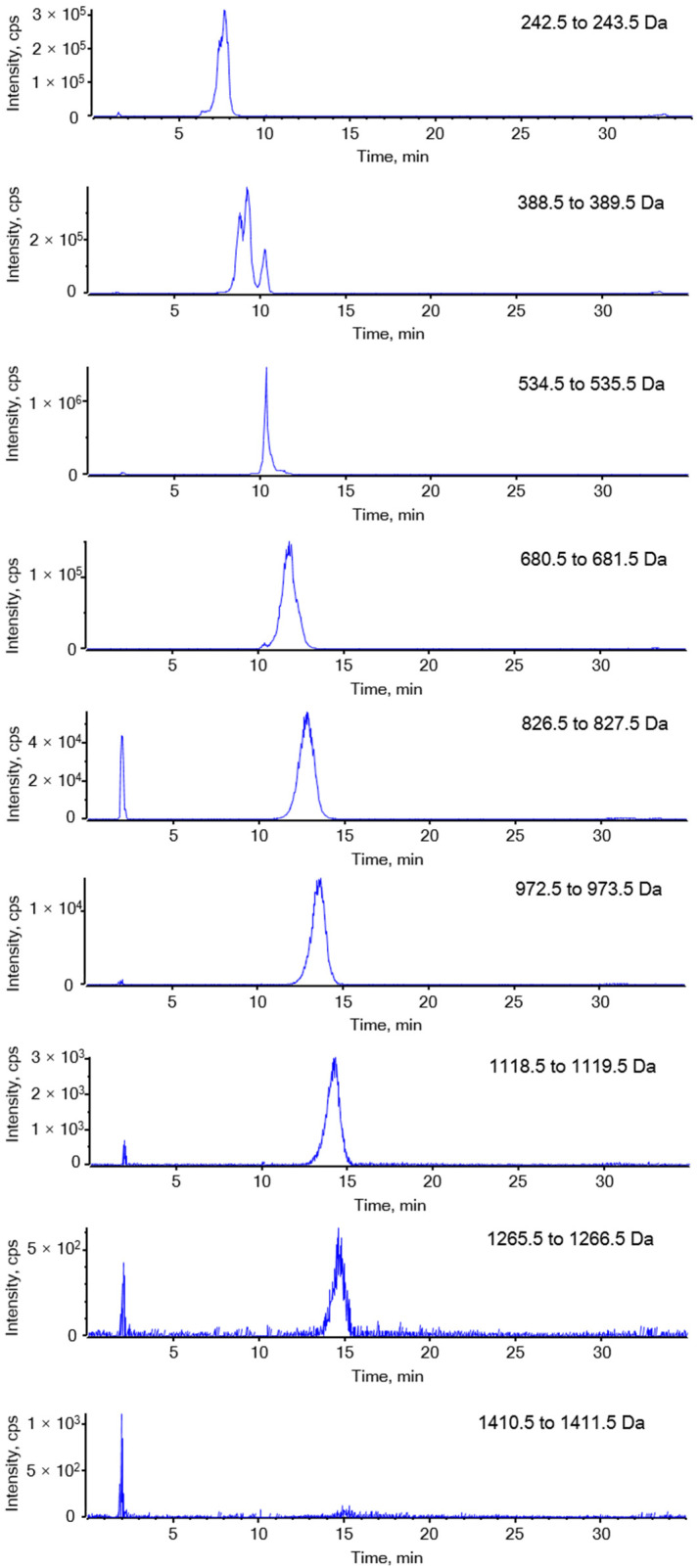
Extracted ion chromatography of DF.

**Figure A3:**
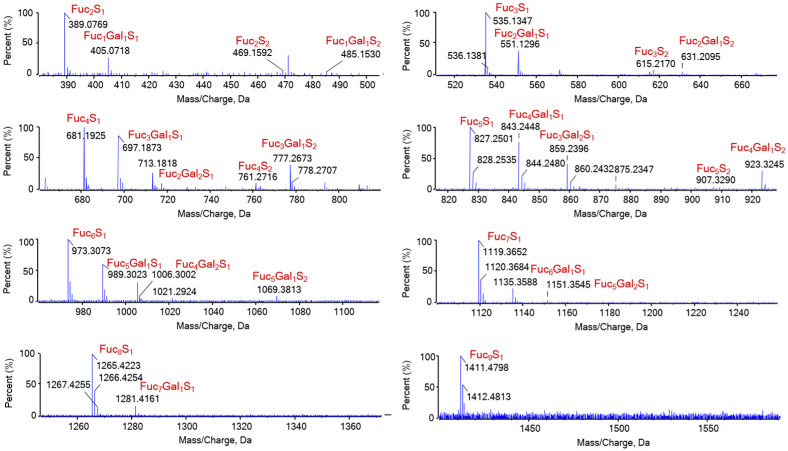
Identified oligosaccharide composition of DF. Fuc_1_Gal_1_S_1_ represent for the oligosaccharide has a composition of one fucose residue, one galactose residue and one sulfate ester group.

LC-MS analysis revealed that DF primarily exists as fucose-oligosaccharides. Extracted ion chromatograms (EICs) were generated for oligomers with degrees of polymerization (DP) ranging from 1 to 9, each containing a single sulfate group, as shown in Figure A2 (Appendix A.2). The MS/MS analysis of ion at *m*/*z* 243 and 389 are presented in Figure 3. The product ion at *m*/*z* 243 provided abundant cleavage information for [Fuc_1_S_1_]^−^. The 2-O-sulfated and 4-O-sulfated forms of the fucose residues were deduced from the ^0.2^X and ^0.2^A fragmentation patterns, respectively, indicating that the sulfated fuco-monosaccharide is a mixture of 2-O- and 4-O-sulfated fucose residues (Figure 3A). The ion at m/z 389 was attributed to the sulfated fuco-disaccharide [Fuc_2_S_1_]^−^. Fragment ions at m/z 164.9841, 225.0050, and 243.0158 resulted from glycosidic bond cleavage, while ions at m/z 138.9691 and 182.9957 confirmed the presence of 2-O and 4-O sulfation. In addition to the main chains, we observed mass increments of 16 Da, which, together with monosaccharide composition results, were interpreted as the substitution of one fucose residue by a galactose unit. Furthermore, a mass increase of 80 Da was attributed to the addition of one sulfate group, while a +96 Da shift corresponded to the simultaneous replacement of one fucose by galactose and the incorporation of one sulfate group (Appendix A.3 Figure A3). The oligosaccharide composition includes two fucose residues, one sulfate ester group [Fuc_2_Gal_1_S_1_]; however, the sequence requires further MS/MS analysis.

### 2.7. Fucoidan Alleviated DSS-Induced UC in Mice

**Figure 4:**
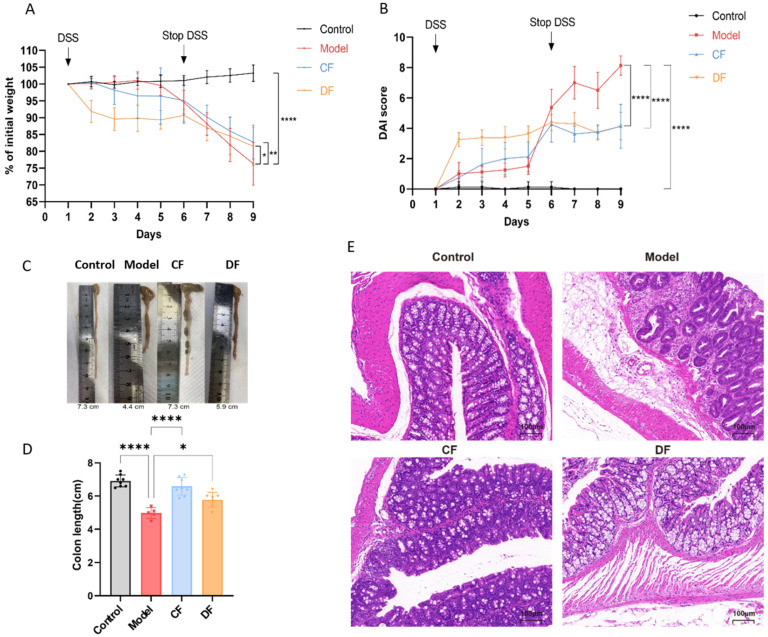
Effects of fucoidan treatment on basic indices of DSS-induced UC in mice. (**A**) Body weight during experiment periods. (**B**) DAI score. (**C**) Image of colon. (**D**) Colon length. (**E**) HE staining image of colon (* *p* < 0.05, ** *p* < 0.01, **** *p* < 0.0001).

The body weight of the control group remained stable, with no statistically significant changes observed. In contrast, the body weight of mice subjected to the DSS intervention significantly decreased (*p* < 0.0001). Following the cessation of the DSS intervention (Days 6–9), the body weight of the model group continued to decline rapidly. Although the body weight of the CF and DF groups also exhibited a continuous decline, the reduction was less pronounced than that of the model group (*p* < 0.01, *p* < 0.05) (Figure 4A). The DAI score of the model group was notably higher than that of the control group (*p* < 0.0001); however, the increased DAI scores were downregulated in both the CF and DF groups compared to the model group (*p* < 0.0001, *p* < 0.0001) (Figure 4B). As shown in Figure 4C, the model group exhibited a significantly shorter colon length compared to the control group. Both CF and DF treatments were effective in reversing this change. The average colon length in the model group was 4.98 ± 0.32 cm, which was significantly shorter than that of the control group, which measured 6.91 ± 0.37 cm (*p* < 0.0001). In contrast, the average colon lengths in the CF and DF groups were 6.59 ± 0.52 cm and 5.77 ± 0.46 cm, respectively, indicating a reversal of the decrease in colon length (Figure 4D). Histological sections stained with hematoxylin and eosin (HE) revealed that the colonic mucosa of the control group mice was intact, exhibiting orderly arranged crypts and no signs of inflammatory cell infiltration or pathological damage. In contrast, the mucosa of the model group displayed severe damage, characterized by epithelial cell shedding, loss of crypt structure, and significant inflammatory cell infiltration. The CF group exhibited occasional mild inflammatory cell infiltration in the submucosa and some localized damage; however, the crypts remained orderly arranged and present in normal quantities. The DF group demonstrated an intact mucosa with well-defined crypt structures and a normal number of goblet cells. Pathological damage to the colon in both the CF and DF groups was alleviated, with the DF group showing a more pronounced protective effect on the colonic mucosa and a reduction in inflammatory infiltration (Figure 4E).

### 2.8. Effects of Fucoidans Treatment on Inflammatory Cytokines in Mouse Serum

**Figure 5:**
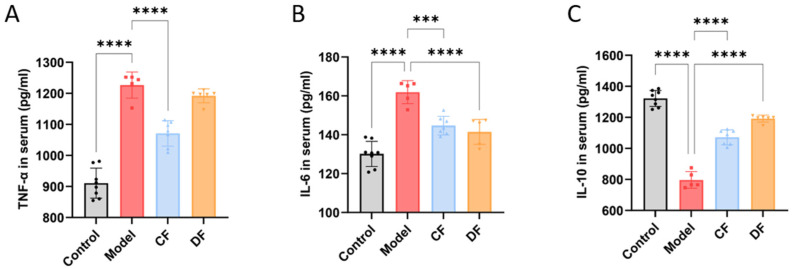
Effects of fucoidans on inflammatory cytokines of DSS-induced UC in mouse serum. (**A**) TNF-α. (**B**) IL-6. (**C**) IL-10. (*** *p* < 0.001, **** *p* < 0.0001).

The levels of pro-inflammatory cytokines TNF-α and IL-6, as well as the anti-inflammatory cytokine IL-10, were measured in the serum of mice from each group (Figure 5). Compared to the control group, the levels of TNF-α and IL-6 in the model group were significantly elevated (*p* < 0.0001), while the level of IL-10 was significantly reduced (*p* < 0.0001). In the CF group, the levels of TNF-α and IL-6 decreased significantly (*p* < 0.0001 and *p* < 0.001, respectively), but there was no significant difference in the level of IL-10 compared to the model group. In the DF group, the level of IL-6 was significantly decreased (*p* < 0.0001), while the level of IL-10 was significantly increased (*p* < 0.0001); however, there was no significant difference in the level of TNF-α compared to the model group.

### 2.9. Effects of Fucoidans Treatment on Gut Microbiota in DSS-Induced UC Mice

**Figure 6:**
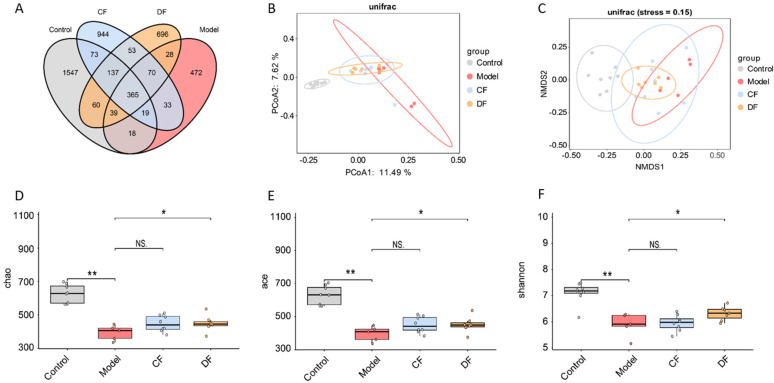
Effects of fucoidans on gut microbiota of DSS-induced UC in mice. (**A**) Venn diagram of ASV. (**B**) Principal coordinate analysis of β-diversity. (**C**) Non-metric multidimensional scaling analysis of β-diversity. (**D**) Chao index analysis. (**E**) ACE index analysis. (**F**) Shannon index analysis. (* *p* < 0.05, ** *p* < 0.01, NS. *p* > 0.05).

A total of 4554 amplicon sequence variants (ASVs) were identified through the analysis of the 16S rRNA gene V3-V4 region sequencing. The control group exhibited a higher number of unique ASVs compared to the other groups, while the model group had the fewest unique ASVs. The unique ASVs in the CF and DF groups fell between those of the control and model groups (Figure 6A). The PCoA plot shows a clear separation between the control and model groups, while the CF and DF groups are distributed between the model and control groups, with data points being more concentrated (Figure 6B). The results of the NMDS analysis are consistent with those of the PCoA; however, the samples from the CF group are more dispersed (Figure 6C). The results of α-diversity, including the Chao, ACE, and Shannon indices, are presented in Figure 6D–F. Compared to the control group, both the Chao and ACE indices in the model group were significantly reduced (*p* < 0.01). The Chao and ACE indices in the DF group exhibited a significant increase compared to the model group (*p* < 0.05), while no significant difference was observed in the CF group. The Shannon index in the model group was significantly lower than that in the control group (*p* < 0.01). The Shannon index in the DF group showed a significant increase compared to the model group (*p* < 0.05), while no significant difference was observed in the CF group.

### 2.10. Effects of Fucoidans Treatment on Taxonomic Levels in DSS-Induced UC Mice

**Figure 7:**
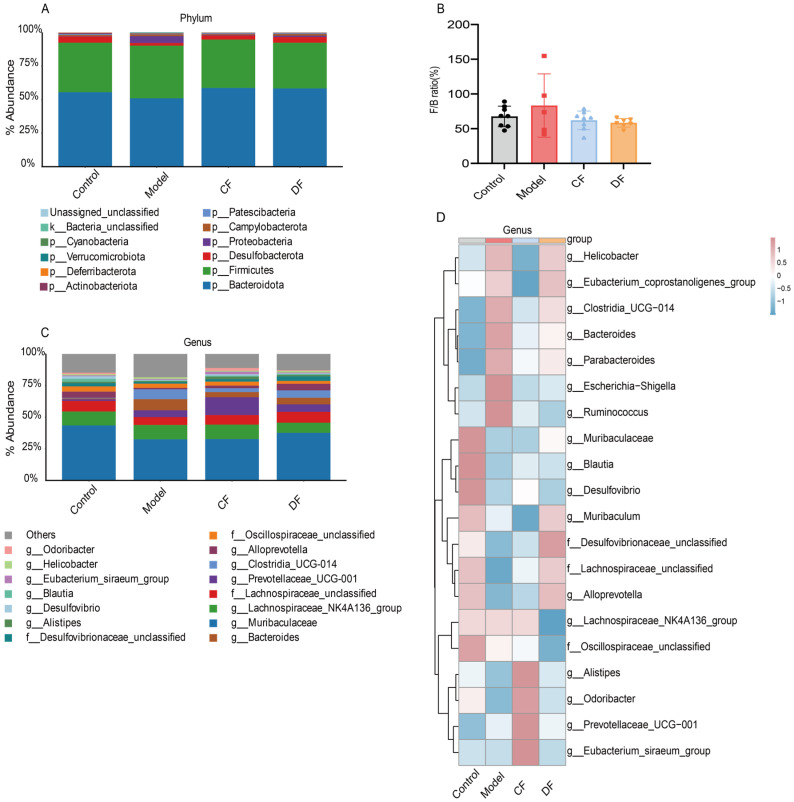
Abundance distribution of gut microbiota at various taxonomic levels. (**A**) Relative abundance bar plot at the phylum level. (**B**) Relative abundance of the Firmicutes to Bacteroidetes ratio. (**C**) Relative abundance bar plot at the genus level. (**D**) Heatmap of the top 20 genera by abundance.

The relative abundances of gut microbiota at the phylum level across the four groups are presented in Figure 7A. All groups were dominated by Bacteroidota and Firmicutes, followed by Desulfobacterota, Proteobacteria, Campylobacterota, and Verrucomicrobiota. Compared with the control group of Bacteroidota (55.51%), DSS-induced UC mice showed a reduction to 50.94%, while fucoidan treatment restored its abundance to 58.69% (CF) and 58.33% (DF). Conversely, Firmicutes increased in the model group (39.26%) compared to the control (36.86%), and fucoidan treatment reversed this trend with CF 35.97% and DF 33.99%, respectively. As shown in Figure 7B, the DSS-induced model group exhibited an elevated Firmicutes-to-Bacteroidetes (F/B) ratio compared with the control group, whereas CF and DF interventions shifted this ratio back toward control values, although no significant differences were observed. The results at the genus level and top 20 abundance genera were selected to construct the heat map shown in Figure 7C,D. In the control group, high relative abundances were observed for *Muribaculaceae*, *Blautia*, *Desulfovibrio*, *Muribaculum*, *Desulfovibrionaceae_unclassified*, *Lachnospiraceae_unclassified*, *Alloprevotella*, and *Odoribacter*. However, these taxa were significantly diminished in mice with DSS-induced UC. Among the fucoidan-treated groups, the CF treatment exhibited no notable effect, whereas the DF treatment effectively restored the abundances of *Muribaculaceae*, *Desulfovibrionaceae_unclassified*, *Lachnospiraceae_unclassified*, and *Alloprevotella*. In contrast, genera such as *Helicobacter*, *Clostridia_UCG-014*, *Bacteroides*, *Parabacteroides*, *Escherichia-Shigella*, and *Ruminococcus* were found at low levels in the control group but were elevated in the model group. The CF intervention reduced the abundances of these taxa, while the DF treatment specifically restored the levels of *Escherichia-Shigella* and *Ruminococcus*.

**Figure 8:**
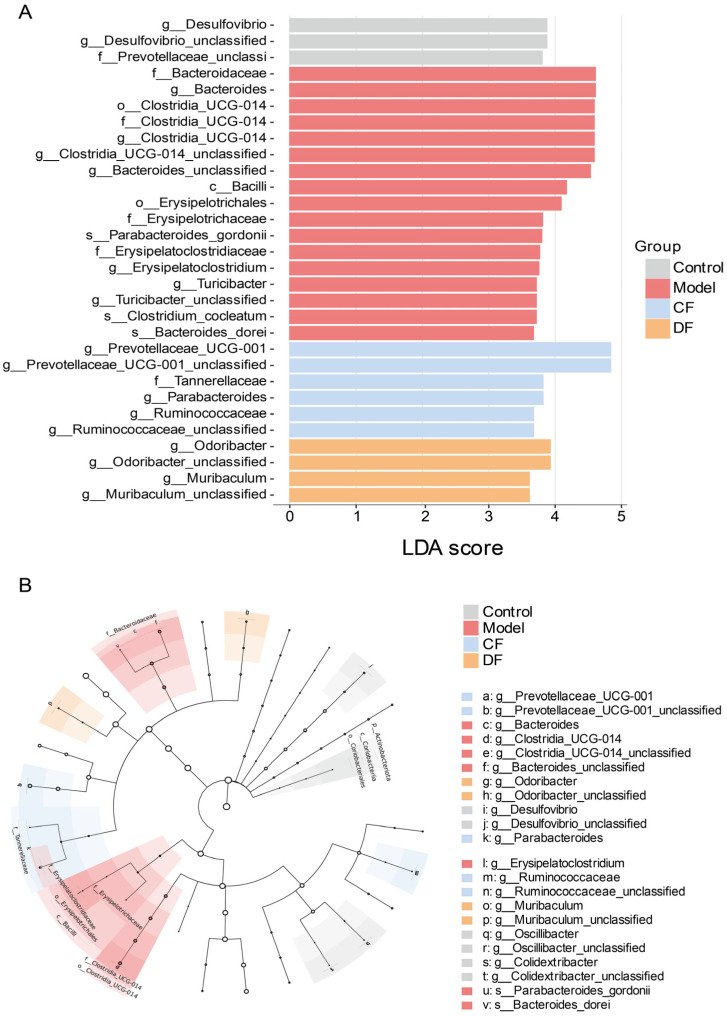
Different microbial analysis between groups (**A**) LEfSe analysis (LDA score > 3.5) (**B**) Taxonomic cladogram.

To elucidate the involvement of gut microbiota in UC mouse models, we conducted Linear Discriminant Analysis Effect Size (LEfSe) to identify taxa that significantly differentiate each experimental group (Figure 8). Applying a discriminant threshold of 3.5, the control group was predominantly characterized by the presence of *Desulfovibrio*, *Desulfovibrio_unclassified*, and *Prevotellaceae_unclassified*. In contrast, following DSS induction, the model group demonstrated an increased abundance at the family level of *Bacteroidaceae*, *Clostridia_UCG-014*, *Erysipelotrichaceae*, and *Erysipelatoclostridiaceae*. At the genus level, enrichment was observed in *Bacteroides*, *Clostridia_UCG-014*, *Bacteroides_unclassified*, *Parabacteroides_gordonii*, *Erysipelatoclostridium*, *Turicibacter*, *Turicibacter_unclassified*, *Clostridium_cocleatum*, *Bacteroides_dorei*. In the groups treated with fucoidan, the CF group exhibited dominance of *Prevotellaceae_UCG-001*, *Tannerellaceae*, *Parabacteroides*, *Ruminococcaceae*, *Ruminococcaceae_unclassified*, whereas the DF group was primarily characterized by *Odoribacter*, *Odoribacter_unclassified*, *Muribaculum*, *Muribaculum_unclassified*.

## 3. Discussion

UC is a chronic, relapsing IBD characterized by persistent inflammation and ulceration of the colonic mucosa. The pathogenesis of UC involves excessive activation of the immune system, leading to the overproduction of pro-inflammatory cytokines, along with dysbiosis of the gut microbiota, which plays a critical role in disease onset and progression [22]. Fucoidan, a naturally derived sulfated polysaccharide, has demonstrated significant anti-inflammatory, immunomodulatory, and gut microbiota-modulating properties. However, the effects of its degree of polymerization and specific structural characteristics on its bioactivity remain poorly understood. This study aims to compare the structural features of native fucoidan and its derivatives and to assess changes in the intestinal microbiota following fucoidan intervention, in order to elucidate the mechanisms and structure–activity relationships underlying fucoidan’s therapeutic effects in colitis.

The structural analysis revealed that the differences between CF and DF include molecular weight distribution and the degree of branching. Sulfate content analysis demonstrated that the overall sulfation levels in both CF and DF are similar; however, LC-MS results indicated that the degree of sulfation in DF does not correlate positively with chain length, with sulfation predominantly occurring at C-2 or C-4 positions. Previous studies have confirmed that the oxygen-radical degradation technique effectively preserve the integrity of the sulfate substituents [23]. FT-IR spectroscopy indicated the presence ofα-linked fucose residues in both CF and DF. Furthermore, methylation analysis, combined with NMR spectroscopy and monosaccharide composition analysis, suggested that the degradation process cleaves not only the glycosidic bonds but also the side chains, leading to a reduced degree of polymerization and diminished side chain complexity in DF compared to CF. Despite these insights, polysaccharide or oligosaccharide sequencing remains challenging for precisely determining branching site substitutions and the location of sulfate esters; therefore, advanced analytical techniques are required to elucidate these subtle structural features within the polysaccharide domain in future research.

CF and DF can mitigate DSS-induced colitis in mouse models, as evidenced by decreased weight loss, reduced incidence of diarrhea and hematochezia, preservation of colon length, restoration of intestinal barrier integrity, and attenuation of colonic tissue damage. Both interventions influenced the diversity, abundance, and composition of the gut microbiota; however, their effectiveness and underlying mechanisms exhibited differences. Colon length serves as a critical biomarker for colitis severity, with inflammation negatively correlating with colon length due to tissue shortening. Histopathological analysis demonstrated that the DF group maintained an intact mucosal layer, well-organized crypt architecture, and normal goblet cell counts. These findings indicate a more effective preservation of the physical barrier which is primarily constituted of Muc2, is secreted exclusively by goblet cells [24].

Inflammatory cytokines play a pivotal role in the progression of UC. Inflamed colonic tissues in UC patients are characterized by an abundance of M1 macrophages, which secrete pro-inflammatory mediators such as TNF-α and IL-6 upon cytokine stimulation [25]. In contrast, M2 macrophages produce anti-inflammatory cytokines, including IL-10, thereby facilitating tissue repair and the resolution of inflammation [26]. While the precise mechanism underlying the enhanced efficacy of CF remains to be fully elucidated, the observed dependence on molecular weight suggests that its larger size may influence fundamental physical barrier protection, as the high molecular weight enables the formation of a viscous colloidal layer along the intestinal mucosa, thereby limiting direct pathogen invasion of the epithelial surface. Future studies specifically designed to investigate this mechanism will be required to determine whether CF confers its superior activity through improved physical barrier protection. Furthermore, CF distinctly modulated inflammation by markedly suppressing TNF-α expression, a cytokine whose overexpression is implicated in exacerbating colonic tissue injury [27].

Gut microbiota diversity is typically assessed through α- and β-diversity, where α-diversity refers to the richness and evenness within individual samples and is commonly measured using indices such as Chao, ACE, and Shannon, and β-diversity captures compositional dissimilarities between different samples with PCoA and NMDS as the statistical techniques. The Chao and ACE indices estimate species richness, with higher values indicating a greater total number of species present. In contrast, the Shannon index evaluates overall diversity, where higher values correspond to increased diversity [28]. Higher β-diversity reflects fewer shared species between microbial communities [29,30]. Samples from the CF and DF treatment groups were positioned intermediate to those of the model and control groups and formed relatively compact clusters, suggesting that both fucoidan variants partially ameliorated gut microbiota composition in UC mice. Notably, samples from the CF group exhibited greater dispersion, indicating increased within-group heterogeneity and implying that the modulatory effects of CF on gut microbiota structure may vary among individual subjects. The F/B ratio is a commonly used biomarker for assessing gut microbial health. Previous studies have shown that patients with UC exhibit an elevated F/B ratio alongside reduced microbial diversity [31,32]. Our findings indicate that these differences were not statistically significant.

At the genus level, DF treatment increased the abundances of *g_Muribaculaceae* and *g_Alloprevotella*, which are involved in dietary fiber degradation and short-chain fatty acid production [33,34]. Additionally, DF elevated the levels of *f_Desulfovibrionace_unclassified* and *f_Lachnospiraceae_unclassified*. *Desulfovibrionaceae* interact with host intestinal epithelial cells and play a crucial role in modulating mucosal function and maintaining gut environmental stability [35], whereas *Lachnospiraceae* are known to effectively prevent colitis [36]. Both CF and DF interventions lead to a reduction in the abundance of *g_Escherichia-Shigella* and *g_Ruminococcus*. *Escherichia-Shigella* is proved to link to intestinal infection and inflammation [37]. Certain strains within *g_Ruminococcus* have been implicated in IBD and obesity; however, in healthy individuals, members of this genus contribute to the degradation of complex carbohydrates and the maintenance of gut homeostasis [38]. *Ruminococcus gnavus* serves as a potential biomarker for multiple disease risks and is a well-established indicator of IBD, exhibiting a positive correlation with disease activity [39]. Additionally, CF treatment may reduce the abundance of several other genera, restoring their levels to those comparable with control groups, particularly *g_Helicobacter*. *Helicobacter pylori* is the most extensively studied species and associated with gastrointestinal disorders [40]. These findings suggest that the modulatory effects of CF and DF on the gut microbiota in UC mice are not solely due to the augmentation of beneficial genera or suppression of harmful ones. Instead, these interventions appear to restore the abundance of genera that deviate from normative levels, thereby contributing to the reestablishment of microbial community balance.

LEfSe analysis further revealed that the species enriched following DSS treatment were consistent with those reported in previous studies [15,41]. *g_Prevotellaceae_UCG-001* was identified as a dominant bacterium in the CF group and has been reported to exhibit a negative correlation with markers of glycolipid metabolism disorders. The proliferation of this genus is associated with the production of substantial quantities of SCFAs, which contribute to the mitigation of host metabolic dysfunctions [42,43]. Another genus, *g_Parabacteroides*, shows decreased abundance in individuals with metabolic syndromes such as obesity and IBD [44]. The specific species within *g_Ruminococcaceae* remain to be further identified. In the DF group, the predominant bacteria were *g_Odoribacter* and *g_Muribaculum*. *Odoribacter* has been reported to be enriched in groups with alleviated UC symptoms [45], and *Odoribacter splanchnicus* contains key components that enhance metabolic processes and provide immune-cell protection against colitis [46]. *Muribaculum* is closely associated with host health, capable of producing SCFAs, regulating intestinal function and immune responses, and is considered to possess probiotic potential [33].

## 4. Materials and Methods

### 4.1. Materials and Reagents

Brown seaweed (*Saccharina japonica*) was collected from Rongchen County (Shandong, China) in June 2018. Dextran sulfate sodium (DSS, Mw 36,000–50,000 Da) was purchased from MP Bio-chemicals (Santa Ana, CA, USA). ELISA kits for interleukin-1β (IL-1β), IL-6, tumor necrosis factor-α (TNF-α) were obtained from Beyotime Biotechnology (Shanghai, China). DEAE Sepharose Fast Flow was obtained from Beijing Ruida Henghui Science & Technology Development Co., Ltd., (Beijing, China). All other reagents utilized in this study were of analytical grade.

### 4.2. Extraction and Purification of Fucoidan (CF)

A dried sample of *S. japonica* was ground into a fine powder using a 60-mesh sieve (250 μm) and then mixed with anhydrous ethanol at a ratio of 1:25 (*w*/*v*). The mixture was stirred at 40 °C for 3 h, followed by filtration under reduced pressure. The residue was collected and air-dried to obtain the defatted sample. The polysaccharide was extracted using 1% calcium chloride (1:30 *w*/*v*) at 100 °C for 3 h and repeated twice. The resulting filtrate was concentrated via rotary evaporation and dialyzed against 3 kDa molecular weight cut-off dialysis membrane. The crude polysaccharide was precipitated by the addition of three volumes of ethanol at 4 °C. The precipitate was then re-dissolved in deionized water to achieve a concentration of 50 mg/mL and stirred for 45 min in a water bath maintained at 40 °C. Subsequently, a 2.5% (*w*/*v*) solution of EDTA was introduced, and the pH was adjusted to 6.0 using a sodium hydroxide. Following this, 2% (*w*/*v*) sodium chloride was added, along with an equal volume of ethanol for precipitation. The precipitation process was repeated for three cycles, followed by purification using Sephadex G-100 to obtain fucoidan (CF).

### 4.3. Preparation of Fucoidan Derivative (DF)

CF was incorporated into a hydrogen peroxide (H_2_O_2_) to achieve a final concentration of 9% and containing 0.1 mM copper acetate. The mixture was then incubated at 60 °C for 5 h. The resulting reaction mixture was subjected to dialysis using a 1 kDa dialysis membrane and then lyophilized. The sample was further purified with DEAE Sepharose Fast Flow resin to obtain the fucoidan derivative (DF).

### 4.4. Chemical Composition Analysis

The total sugar content was determined using the phenol-sulfuric acid method [47], while the protein content was measured by the Bradford assay [48]. Uronic acid content was assessed using the carbazole colorimetric method [49], and sulfate content was determined through the chlorination method [50].

### 4.5. Molecular Weight Determination

The molecular weight was determined using a Waters 2695 HPLC system equipped with an evaporative light scattering detector (ELSD) (Waters Corporation, Milford, MA, USA). The system was connected to a TSK G5000 PWxl column (7 μm, 7.8 × 300 mm), TSK G3000 PWxl column (7 μm, 7.8 × 300 mm) G-Oligo-PW (7 μm, 7.8 × 150 mm) (Tosoh Bioscience, Tokyo, Japan). The elution was performed with deionized water at a flow rate of 0.5 mL/min. Samples were prepared at a concentration of 10 mg/mL, and injection volume was set at 10 μL. The ELSD settings were configured as follows: detector gain at 100, gas pressure at 30.0 psi, nebulizer operating in cooling mode, and drift tube temperature set to 50 ± 25 °C. Dextran of varying molecular weights was used as standards. By plotting retention time on the horizontal axis and the logarithm of the average molecular weight of the standard samples on the vertical axis, create the standard curve.

### 4.6. Monosaccharide Composition Analysis

A pre-column derivatization method using 1-phenyl-3-methyl-5-pyrazolone (PMP) in conjunction with the LC-MRM-MS method was employed to determine the monosaccharide composition [51]. Samples (2 mg) were dissolved in 1 mL of 4 M trifluoroacetic acid (TFA) and subjected to hydrolysis at 110 °C for 4 h in a sealed glass container. Subsequently, the mixture was dried under a nitrogen atmosphere and then re-dissolved in 0.2 mL methanol, followed by drying three times to eliminate TFA. The dried sample was reconstituted in 100 μL of 0.1 M sodium hydroxide and adjusted to a final volume of 1 mL with water. The hydrolyzed sample (400 μL) was combined with 450 μL of 0.3 M sodium hydroxide and 450 μL of 0.5 M PMP for derivatization at 70 °C for 30 min. Then, 450 μL of 0.3 M hydrochloric acid was added to neutralize the reaction and chloroform was employed to remove PMP. The resulting supernatant after centrifugation was subjected for further analysis. The LC-MS was performed on an ExionLC system coupled with a TripleQuad MS in MRM mode (AB SCIEX, Framingham, MA, USA). A Waters ACQUITY Premier HSS T3 column (1.8 μm, 2.1 × 100 mm) was utilized with a flow rate at 0.3 mL/min. The results compared the retention time and peak area with those of the monosaccharide standards.

### 4.7. Glycosidic Linkage Analysis

De-sulfated sample (10 mg) was mixed with 5 mL of DMSO and sonicated under nitrogen atmosphere until fully dissolved. Powdered sodium hydroxide (100 mg) was then added to the solution and allowed to react for 1 h. Then iodomethane (1 mL) was introduced and reacted for 1 h in the dark. The reaction was terminated by adding 1 mL of water. The methylated samples were extracted with an equal volume of chloroform and repeated for three times. Subsequently, 2 M TFA (2 mL) was added to the methylated samples for hydrolysis at 110 °C for 4 h. The TFA was removed using methanol, and the resulting samples were re-dissolved in 1.5 mL of water. Sodium borohydride (20 mg) was added and allowed to react for 2.5 h. The reaction was neutralized with acetic acid and dried under nitrogen. The sample was then treated with 0.5 mL of pyridine and glacial acetic anhydride in a 1:1 ratio and allowed to react for 30 min at 100 °C. The sample was extracted twice with chloroform and then concentrated for further analysis. The GC-MS was conducted using an Agilent 7890A–7000B system equipped with a DB-17ms column (30 m × 250 μm × 0.25 μm). The initial temperature was set at 100 °C, held for 2 min, then increased to 220 °C at a rate of 5 °C/min, and held for 15 min. The split ratio was 50:1, and the flow rate was maintained at 1 mL/min.

### 4.8. Fourier-Transform-Infrared (FT-IR) Analysis

The polysaccharide samples (1 mg) were compressed into a circular form using potassium bromate (99 mg) and subsequently analyzed using a FT-IR spectrometer (Thermo Fisher Scientific, Waltham, MA, USA) within the frequency range of 4000 to 400 cm^−1^.

### 4.9. NMR Analysis

Samples were dissolved in 0.4 mL D_2_O and lyophilized, repeated twice. Subsequently, ^1^H and ^13^C NMR spectroscopy analysis were performed on a Bruker Avance 500 MHz spectrometer (Bruker, Billerica, MA, USA).

### 4.10. Oligosaccharide Mapping of DF

DF was analyzed using a Q-TOF mass spectrometer coupled with a UPLC system (AB SCIEX, Framingham, MA, USA). Separation was performed on a GlycanPac AXH-1 column (2.1 × 150 mm) maintained at 40 °C. Mobile phase A consisted of 5 mmol/L ammonium acetate, and mobile phase B was acetonitrile. The flow rate was set at 0.25 mL/min with the following gradient: 0~5 min, 20%A, 5~25 min, increased from 20% to 80% A and held for 5 min; then immediately returned to 20% A and held for an additional 5 min. Mass spectrometry was conducted in negative-ion mode with source parameters as follows: curtain gas, 35 psi; ion source gas 1:55 psi; ion source gas 2:55 psi; temperature: 600 °C; spray voltage: −4500 V; and scan range: 100~2000 Da.

### 4.11. Animals and Treatments

Thirty-two male C57BL/6 mice, aged 8 weeks, were purchased from Hangsi Biotechnology (Hangzhou, China) and housed in a specific pathogen-free (SPF) animal laboratory. The environmental conditions were maintained at a temperature of 24 ± 2 °C, with a relative humidity of 50%, and a 12 h light-dark cycle. After a one-week acclimation period, the mice were randomly assigned to four groups (*n* = 8). The control group received oral gavage of normal saline at a dose of 200 mg/kg body weight per day and had unrestricted access to drinking water. The model, CF, and DF groups received oral gavage of normal saline, CF, and DF at a dose of 200 mg/kg body weight per day, respectively, along with a 2% DSS solution for the first five days, followed by drinking water (Table 3). Body weight was recorded daily, and blood samples were collected from the angular vein. Serum was separated and collected immediately by centrifugation at 3000 rpm for 20 min at 4 °C. All mice were sacrificed using ether anesthesia after fasting overnight. Colon tissues were harvested post-dissection for further examination. All procedures were conducted in accordance with the protocols approved by the Ethics Committee of Zhejiang University of Technology (approval no. 20220311040).

### 4.12. Assessment of the Disease Activity Index

The Disease Activity Index (DAI) score is determined by assessing changes in body weight, stool consistency, and the presence of bloody stool, which collectively reflect the overall disease activity. Throughout the experiment, the body weight, stool consistency, and occurrence of bloody stool in mice were recorded daily prior to gavage and subsequently scored according to the DAI scoring criteria [52].

### 4.13. Histopathological Assessment and Immunofluorescence Staining

The length of the colon was measured immediately after the dissection of the mice. Then, the tissues were embedded in paraffin for slices (4 μm) and stained with hematoxylin and eosin (H&E). All slices were examined for the extent of colonic damage, including epithelial cell shedding, destruction of crypt structures, and infiltration of inflammatory cells, using an optical microscope (DM 2000 LED, Leica Microsystems, Wetzlar, Germany).

### 4.14. Detection of Inflammatory Factors in Serum

Serum samples were allowed to naturally thaw for 30 min at room temperature and then centrifuged at 3000 rpm for 20 min at 4 °C. Inflammatory cytokines for TNF-α, IL-6, and IL-10 in serum were measured using ELISA kits according to the manufacturer’s instructions.

### 4.15. 16S rRNA Sequencing of Colonic Contents

Microbial DNA was extracted from the colonic content using the KAPA HiFi HotStart Ready Mix Kit according to the manufacturer’s protocol. The V3-V4 region of the bacterial 16S rRNA gene were amplified with primers B341F (5′-CCTACGGGNGGCWGCAG-3′) and B785R (5′-GACTACHVGGGTATCTAATCC-3′) using a thermocycler PCR system (T100 Thermal Cycler, Bio-Rad, Hercules, CA, USA). The amplicons were purified using AMPure XP Beads, quantified using the KAPA Illumina Library Quantification Kit, and sequenced by Hangzhou Kaitai Biotechnology Co., Ltd. (Hangzhou, Zhejiang, China) on its Illumina HiSeq PE250 platform. Optimized sequences were clustered in the operational taxonomic units (OTUs) for further bioinformatics analysis.

### 4.16. Statistical Analysis

The data were processed using GraphPad Prism 9.5 (GraphPad Software, San Diego, CA, USA) and presented as mean ± standard deviation (SD). One-way analysis of variance (ANOVA) followed by Dunnett’s multiple comparison tests was performed for different analysis. The significance of the results was indicated as follows: * *p* < 0.05, ** *p* < 0.01, *** *p* < 0.001, **** *p* < 0.0001. Linear discriminant analysis Effect Size (LEfSe) was conducted to identify biomarkers and distinct features, the Kruskal–Wallis rank sum test was used to evaluate differences in absolute abundance between groups, and linear discriminant analysis (LDA) was applied to estimate the impact of distinguishing microbial communities on the observed differences between groups.

## 5. Conclusions

We conducted a comparative analysis of the structural characteristics of CF and its degraded derivative, DF, as well as their respective efficacies in mitigating UC. The structural difference between CF and DF included degree of polymerization and branching. The side chain of DF partially degraded during H_2_O_2_ treatment compared to CF, and molecular weight decreased from 582 kDa to 2.3 kDa. The monosaccharide composition and degree of sulfation exhibited similar ratios. Both compounds demonstrated therapeutic potential in alleviating UC symptoms in mouse models. However, their mechanisms of action appear to differ. CF exhibited superior efficacy in reducing weight loss and preserving colon length, whereas DF showed a greater capacity for protecting the colonic mucosa and inhibiting inflammatory cell infiltration. Both CF and DF can alter the gut microbiota composition in UC mice. The dominant species associated with CF are *Prevotellaceae_UCG-001*, *Parabacteroides*, and *Ruminococcaceae* while in DF, the predominant species are *Odoribacter* and *Muribaculum.*

## Figures and Tables

**Table 1:** Chemical properties of CF and DF.

Sample	Total Sugar (%)	Protein (%)	Uronic Acid (%)	Sulfate Content (%)	Mw (kDa)	Monosaccharide (%)						
						Man	GlcA	GalA	Glc	Gal	Xyl	Fuc
CF	49.6 ± 1.03	2.77 ± 0.13	1.78 ± 0.11	20.2 ± 2.03	582	7.57	2.12	1.85	6.07	26.23	0.30	55.86
DF	74.0 ± 1.58	0.59 ± 0.08	1.36 ± 0.09	23.3 ± 0.95	2.3	3.23	1.62	1.49	6.58	25.08	0.16	61.85

**Table 2:** Methylation analysis of CF and DF.

PMAA	Linkage Pattern	Major Mass Fragments (Da)	Molar Ratios (%)	
			CF	DF
2,3,4-Me3-Fuc	T-Fuc*p*	59, 69, 72, 88, 89, 102, 115, 118, 131, 160, 162, 175	5.74	10.25
2,4-Me2-Fuc	1,3-Fuc*p*	43, 57, 71, 85, 89, 101, 117, 131, 159, 173, 189	47.25	62.28
2-Me-Fuc	1,3,4-Fuc*p*	43, 55, 71, 87, 97, 99, 117, 129, 141, 157, 173, 175, 231	14.62	5.82
2,4,6-Me3-Gal	1,3-Gal*p*	59, 87, 101, 118, 129, 143, 161, 174, 202, 217	16.28	15.21
4,6-Me2-Gal	1,2,3-Gal*p*	43, 57, 95, 109, 129, 145, 161, 189, 207, 261	10.31	4.57
2,4,6-Me3-Man	1,3-Man*p*	43, 57, 71, 85, 113, 141	3.23	nd
2,4,6-Me3-Glc	1,3-Glc*p*	59, 87, 101, 118, 129, 157, 202, 234	2.57	1.87

**Table 3:** Design and grouping of mouse experiments.

	7 Days	Day 1–5	Day 6–9	Day 10
Control	Adaptation period	Water		Sacrifice, collect blood, colon tissues
		gavage with 200 mg/kg normal saline		
Model		2% DSS	water	
		gavage with 200 mg/kg normal saline		
CF		2% DSS	water	
		gavage with 200 mg/kg CF		
DF		2% DSS	water	
		gavage with 200 mg/kg DF		

## Data Availability

The original contributions presented in this study are included in the article. Further inquiries can be directed to the first/corresponding authors (yanleiyu@zjut.edu.cn; hongw@zjut.edu.cn).

## Abbreviations

The following abbreviations are used in this manuscript:

UC	Ulcerative colitis
IBD	Inflammatory bowel disease
DSS	Dextran sulfate sodium
PMP	1-phenyl-3-methyl-5-pyrazolone
TFA	Trifluoroacetic acid
DAI	Disease Activity Index
LDA	Linear discriminant analysis
ASVs	Amplicon sequence variants
LEfSe	Linear Discriminant Analysis Effect Size
